# Apoptotic mechanisms in T47D and MCF-7 human breast cancer cells

**DOI:** 10.1038/sj.bjc.6600541

**Published:** 2002-10-07

**Authors:** L M Mooney, K A Al-Sakkaf, B L Brown, P R M Dobson

**Affiliations:** Institute for Cancer Studies, Division of Genomic Medicine, Medical School, University of Sheffield, Sheffield S10 2RX, UK; Academic Unit of Endocrinology, Division of Genomic Medicine, Medical School, University of Sheffield, Sheffield S10 2RX, UK

**Keywords:** apoptosis, breast cancer, caspase-3, staurosporine

## Abstract

To investigate the mechanisms underlying apoptosis in breast cancer cells, staurosporine was used as an apoptotic stimulus in the human breast cancer cell lines MCF-7 and T47D. Staurosporine induced dose and time dependent increases in DNA fragmentation which was abrogated by z-VAD-fmk. MCF-7 cells did not express caspase-3, suggesting that DNA fragmentation occurred in the absence of caspase-3 and that other caspases may be involved. Staurosporine induced DEVDase activity in T47D cells suggesting the involvement of caspase-3 and/or caspase-7, yet there was no DEVDase activity in MCF-7 cells, probably ruling out the involvement caspase-7. However, staurosporine induced the cleavage of pro-caspase-6 in MCF-7 cells, but not in T47D cells. Caspase dependent PARP cleavage was detected in MCF-7 cells at 3 h, whereas only partial PARP cleavage was detected in T47D cells and then only after 24 h. Moreover, staurosporine led to cytochrome *c* release at 2 h in MCF-7 cells and 6 h in T47D cells. In addition, a time dependent and caspase-independent reduction of the mitochondrial transmembrane potential was observed; which appeared to occur after the release of cytochrome *c*. Translocation of Bax from the cytosol to mitochondria was observed in both cell types, and this preceded cytochrome *c* release in both T47D and MCF-7 cells. Apoptotic events in both cell types differ temporally, involving activation of different caspases and mitochondrial changes.

*British Journal of Cancer* (2002) **87**, 909–917. doi:10.1038/sj.bjc.6600541 www.bjcancer.com

© 2002 Cancer Research UK

## 

Apoptosis is an essential process in both multi-cellular development, and in maintaining cellular homeostasis. Moreover, failure of damaged cells to undergo apoptosis contributes to the progression of cancer as it allows the persistence of DNA damaged cells. It is therefore of interest to understand apoptotic signalling pathways in different cancer types. Apoptosis is morphologically defined by cell shrinkage, membrane blebbing, chromatin condensation and formation of apoptotic bodies. Central to the apoptotic process is a family of cysteine proteases termed caspases which are crucial effectors of this cell death process. Caspases exist in the cell as inactive pro-enzymes that can be activated either by auto-catalytic processing or by being activated by another caspase (Earnshaw *et al*, 1999). These proteases can be classified into two groups: ‘initiator’ caspases e.g. caspases-8 and -9, which have long prodomains at the NH_2_-termini and have the ability to be self-cleaved on oligomerisation, and ‘effector’ caspases e.g. caspases-3, -6 and -7 that have short prodomains and can be activated by initiator caspases or by activated effector caspases (Thornberry and Lazebnik, 1998). Once activated by apoptotic stimuli, caspases contribute to the morphological and biochemical changes of apoptosis by catalysing the proteolysis and disabling of numerous key structural and regulatory proteins within the cell such as, poly-ADP-ribose polymerase (PARP), gelsolin, cytokeratins and DNA fragmentation factor 45 kDa (DFF45) (Nunez *et al*, 1998).

The Bcl-2 family of anti- (Bcl-2, Bcl-x_L_, Mcl-1) and pro-apoptotic (Bax, Bad, Bid) proteins are also important in the regulation of cell death. Bcl-2 family members have the ability to form homo- and hetero-dimers and the ratio of anti- and pro-apoptotic proteins may determine the fate of the cell (Reed, 1998). These proteins can be either membrane bound or cytosolic. Bcl-2 has been shown to be localised to the endoplasmic reticulum, mitochondrial membrane and the nuclear envelope (Krajewski *et al*, 1993), whereas other members such as Bax and Bid are mainly cytosolic. However on induction of apoptosis, localisation of some Bcl-2 family members alters. For example, Bax has been shown to translocate from the cytosol to the mitochondrial membrane after treatment with an apoptotic stimulus (Zhang *et al*, 1998), and cause subsequent cytochrome *c* release and caspase activation. Due to their diversity in function these proteins constitute an important point of regulation in the apoptotic pathway.

An alteration in mitochondrial function has been demonstrated to play a key role in the effector phase of the apoptotic pathway. A reduction in the mitochondrial transmembrane potential (ΔΨm) and release of the mitochondrial protein cytochrome *c* is often observed during apoptosis (Zamzami *et al*, 1995; Liu *et al*, 1996). On induction of apoptosis cytochrome *c* is released from the intermembrane space into the cytoplasm where it leads to activation of caspases and subsequent cell death (Liu *et al*, 1996). Mitochondrial transmembrane potential breakdown is thought to be mediated by the opening of a large conductance channel known as the mitochondrial permeability transition pore (PTP) (Zamzami *et al*, 1996). Studies have shown cytochrome *c* release to occur in the absence of, or to precede ΔΨm disruption (Bossy-Wetzel *et al*, 1998; Goldstein *et al*, 2000), however the exact mechanism of cytochrome *c* release remains uncertain. The anti-apoptotic effect of Bcl-2 and Bcl-x_L_ has been shown to involve the prevention of cytochrome *c* release and ΔΨm loss whereas pro-apoptotic Bax can induce these mitochondrial changes (Decaudin *et al*, 1997; Yang *et al*, 1997; Jurgensmeier *et al*, 1998).

The order of apoptotic events appears to vary widely depending on the cellular background and the apoptotic stimulus. It is, therefore, important to understand the molecular components of the death machinery of the cell to aid the establishment of appropriate therapeutic intervention. Indeed, caspase knock-out studies have shown that not all caspases are essential for apoptosis (Zheng *et al*, 1999). Caspase-3 has been shown to play a central role in key apoptotic events such as DNA fragmentation and membrane blebbing, and caspase-3 null mice die perinatally and display hypercellularity (Woo *et al*, 1998). MCF-7 breast cancer cells also lack caspase-3 protein, yet they remain responsive to many apoptotic stimuli suggesting functional redundancy within the caspase family (Janicke *et al*, 1998a). The present study was aimed at investigating the intracellular apoptotic signalling mechanisms in T47D (caspase-3 positive) and MCF-7 (caspase-3 negative) breast cancer cells in response to the general protein kinase inhibitor and apoptotic stimulus, staurosporine (STS). We found that, in these two similar solid tumour types, differences exist in the mechanisms by which they undergo apoptosis. Our results show that the mechanism of cell death was kinetically different with events occurring earlier in MCF-7 cells, than in T47D cells. In addition, this involved release of cytochrome *c*, a reduction in the mitochondrial transmembrane potential (ΔΨm) and activation of different aspects of the caspase cascade.

## MATERIALS AND METHODS

### Materials

Staurosporine (STS) and z-VAD-fmk were from Alexis Biochemicals (Nottingham, UK). *E. Coli*. DNA polymerase 1 and all culture media and sera were obtained from Gibco BRL (Paisley, UK). The monoclonal mouse antibody against Bcl-2, the polyclonal antibody against Bcl-x_L_ and the polyclonal antibody to caspase-6 were obtained from Santa Cruz Biotechnology Inc., (California, USA). The polyclonal rabbit antibody to Bax was purchased from TCS Biologicals Ltd., (Buckingham, UK). PARP, Bak and cytochrome *c* antibody were from Pharmingen (Oxford, UK). The CaspACE Assay System, Colorimetric was from Promega, (Southampton, UK). The Cell death ELISA kit and protease cocktail tablets were from Boehringer Mannheim, (East Sussex, UK). The ECL system was obtained from Amersham, (Buckinghamshire, UK).

### Cell culture

T47D (ductal carcinoma) and MCF-7 (adenocarcinoma) human breast cancer cell lines were maintained in Dulbecco's Modified Eagle's Medium (DMEM) supplemented with 10% (v v^−1^) foetal calf serum (FCS) and 2 mM glutamine. Cells were maintained at 37°C in a humidified atmosphere of air/CO_2_ (19 : 1). Cells were incubated with 1% FCS DMEM media during experimentation for the times indicated.

### *In situ* nick translation (ISNT)

Cells were incubated with staurosporine for different times in the presence or absence of z-VAD-fmk. Adherent and detached cells were pooled and were fixed in 1% paraformaldehyde and permeabilized in 70% ethanol at −20°C. Cells were then washed in PBS and incubated at room temperature for 4 h with nick translation buffer (50 mM TRIS, 10 μg ml^−1^ bovine serum albumin (BSA), 2.5 mM MgCl_2._6H_2_O, 10 mM β-mercaptoethanol) containing *E. Coli* DNA polymerase (1 unit), 0.2 nM biotin-dUTP and 0.2 nM each of dATP, dGTP and dCTP. Samples were washed and resuspended in staining buffer (600 mM NaCl, 60 mM sodium citrate, 0.1% Triton X-100, 5% non-fat dry milk (Marvel)) supplemented with 2.5 μg ml^−1^ avidin-FITC. The samples were then washed and analysed by Flow Cytometry. Aliquots of certain samples (100 μl) were stained with 1 μg ml^−1^ 4,6-diamidino-2-phenylindole (DAPI), cytospun onto slides and visualised under a fluorescent microscope using IP Lab Spectrum Software (Signal Analytics, Vienna, VA, USA) for apoptotic nuclear morphology.

### DNA fragmentation ELISA (Boehringer Mannheim)

This assay measures cytoplasmic histone-bound DNA fragments (mono- and oligonucleosomes) which are generated during apoptosis (Boehringer Mannheim). The enrichment of nucleosomes in the cytoplasm of treated cells was expressed as fold induction in apoptosis compared to untreated controls. Cells (1×10^5^) were incubated with 1 μM STS, washed in PBS and then lysed in lysis buffer for 30 min. The supernatant (cytoplasmic extract) was recovered and the assay was performed according to the manufacturer's protocol.

### Annexin-V-FITC assay (BioSource International Inc)

Cells (5×10^5^) were treated with 1 μM STS for times indicated. Adherent and detached cells were pooled, washed and then resuspended in binding buffer (buffer 10 mM HEPES/NaOH, pH 7.4, 140 mM NaCl, 2.5 mM CaCl_2_) and annexin-V-FITC antibody (5 μl), mixed and incubated for 10 min at room temperature. Cells were washed and resuspended in binding buffer containing propidium iodide (PI) (10 μl) to give a final concentration of 1 μg ml^−1^ PI prior to analysis by Flow Cytometry. Bivariant analysis of FITC-fluorescence (FL-1) and PI-fluorescence (FL-3) gave different cell populations where FITC −ve and PI −ve were designated as viable cells; FITC +ve and PI −ve phenotype as apoptotic cells, and FITC +ve and PI +ve as late apoptotic or necrotic cells.

### Caspase activity assay (Promega)

The CaspACE Assay System was utilised to detect DEVDase (caspase-3/7) activity. Cells were treated with 1 μM STS and lysed in lysis buffer from the kit. Protein content was determined and lysates incubated with Ac-DEVD-pNA for 4 h at room temperature. The reaction product was detected at 405 nm using an Athos 2001 automated plate reader (Athos Labtec Instruments, UK).

### Cytofluorometric analysis of mitochondrial transmembrane potential (ΔΨm)

To measure ΔΨm, cells (5×10^5^) were incubated with 40 nM 3,3-dihexyloxacarbocyanine iodide (DiOC_6_(3)) for 20 mins at 37°C. As a positive control to show complete depletion of ΔΨm, the mitochondrial uncoupler, carbonyl cyanide m-chlorophenylhydrazone (CCCP at 50 μM) was used. Propidium Iodide (PI) was added prior to FACS analysis as a measure of cell viability. Fluorescence of the total cell population was measured with DiOC_6_(3) at green fluorescence (FL-1) and PI at red fluorescence (FL-3) by FACScan using CellQuest software (Becton Dickinson).

### Western blotting

Cells were treated with 1 μM STS for different periods of time, then washed in ice cold PBS and lysed for 20 min on ice in lysis buffer (50 mM Tris.HCl, pH 7.4, 150 mM NaCl, 1% (v v^−1^) NP-40, 0.625% sodium deoxycholate, 1 mM NaF, 1 mM Na_3_VO_4_, 1 protease cocktail tablet (Boehringer Mannheim)). Equal amounts of total protein (30 μg) were subjected to 14% (Bcl-2 family proteins, cytochrome *c* and cytochrome *c* oxidase), 8% (Poly ADP-Ribose Polymerase (PARP)) or 10% (caspase-6) SDS–PAGE followed by Western transfer onto a PVDF membrane. Membranes were incubated with antibodies against Bcl-2, Bax, Bak and Bcl-x_L_, PARP, caspase-6 or cytochrome *c* and detected with the appropriate species-specific secondary HRP-conjugated antibody. Proteins were detected using the ECL system (Amersham, UK).

## RESULTS

### Staurosporine induced apoptosis in T47D and MCF-7 human breast cancer cells

**Figure 1:**
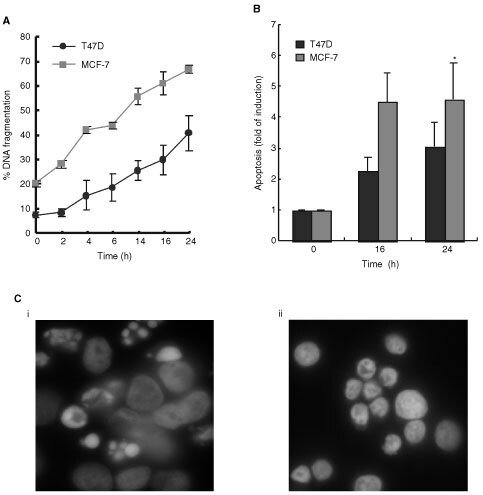
Staurosporine induced apoptosis in T47D and MCF-7 human breast cancer cells. (**A**) Cells were treated with 1 μM STS for the times indicated and apoptosis was determined by DNA fragmentation (ISNT) on fixed and permeabilised cells. Data are presented as per cent DNA fragmentation: error bars represent the mean±s.e.m. of three independent experiments. (**B**) Cells were treated with 1 μM STS for the times indicated, lysates were analysed by ELISA according to the manufacturer's instructions. Fold induction in apoptosis is expressed as amount of cytoplasm DNA-histone complexes in treated cells compared to untreated controls. Error bars represent the mean±s.e.m. of three independent experiments where * indicates statistically significant increase in apoptosis compared to untreated control (0 h), (ANOVA: *F*=5.44, *P*<0.05, d,f 2,9. Tukey multiple comparison test, *P*<0.05). (**C**) Apoptotic nuclear morphology. (i) T47D cells (ii) MCF-7 cells. Cells were treated with 1 μM STS for 16 h, then washed in PBS, fixed, permeabilised and stained with 1 μg ml^−1^ DAPI. Cells were then analysed by fluorescent microscopy.

**Figure 2:**
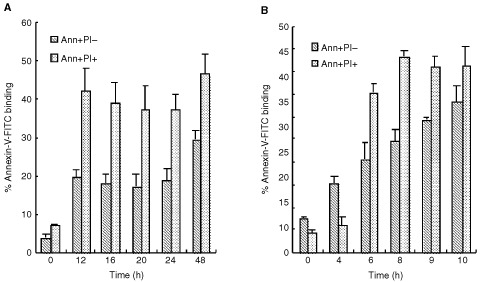
Staurosporine induced a time dependent increase in annexin-V binding in (**A**) T47D and (**B**) MCF-7 cells. Both T47D and MCF-7 cells were treated with 1 μM STS for the times indicated. Cells were washed in PBS and stained with annexin-V-FITC and PI and analysed by Flow Cytometry. Cells staining annexin-V-FITC+/PI- were considered apoptotic; cells staining annexin-V-FITC+/PI+ were classified as late apoptotic/necrotic. Data are presented as per cent of cells in each classification. Error bars represent the mean±s.e.m. of three independent experiments.

Studies of apoptosis, as determined by DNA fragmentation (ISNT), demonstrated that T47D and MCF-7 cells, when exposed to STS (0.2–1 μM), exhibited a concentration-dependent increase in apoptosis, with maximal effects for both cells being at 1 μM STS (data not shown). Apoptosis was also a time-dependent process. When exposed to 1 μM STS, a significant increase in cells displaying DNA fragmentation was evident at 14 h in T47D cells, yet apoptotic changes were detected as early as 4 h in MCF-7 cells (Figure 1A

). We further examined DNA fragmentation by cell death ELISA. Results presented in Figure 1B show a time-dependent increase in the presence of mono- and oligo-nucleosomes similar to that observed with ISNT in both cell types, with MCF-7 cells exhibiting greater levels of apoptosis. The apoptotic effect was confirmed by nuclear morphology (Figure 1C), with both cell types showing smaller nuclei and chromatin condensation resulting in the formation of crescent shapes around the periphery of the nucleus. However, T47D cells displayed apoptotic body formation although this was absent from MCF-7 cells (Figure 1C). Furthermore, plasma membrane changes, such as exposure of phosphatidyl-serine (PS) residues to the outer surface of the plasma membrane were evident during STS-induced apoptosis. STS induced an increase in binding of annexin-V in MCF-7 cells after 4 h of treatment and continued to increase, however this occurred later in T47D cells where significant annexin-V binding was achieved 12 h after 1 μM STS (Figure 2

). No changes in annexin-V binding were detected before 12 h in T47D cell (data not shown). This demonstrates that plasma membrane changes probably occur just prior to DNA fragmentation in T47D cells however, in MCF-7 cells these changes appear to occur simultaneously.

**Figure 3:**
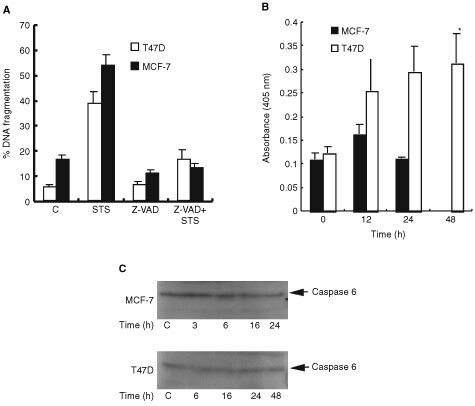
Staurosporine induced apoptosis activation of the caspase cascade in both MCF-7 and T47D cells. (**A**) Cells were pre-incubated with 100 μM z-VAD-fmk (Z-VAD) for 1 h prior to the addition of 1 μM STS for 16 h. Cells were fixed, permeabilised and DNA fragmentation detected by ISNT. Error bars represent the mean±s.e.m. of three independent experiments. (**B**) Cells were treated for different time periods with 1 μM STS. Cell lysates were prepared and incubated with Ac-DEVD-pNA (caspase-3/7). The reaction products were measured at 405 nm after a 4 h incubation. Error bars represent the mean±s.e.m. of four independent experiments where * indicates statistically significant increase in DEVDase activity compared to untreated control (0 h), (ANOVA: *F*=3.38, *P*<0.05, d,f 3,12. Tukey multipe comparison test, *P*<0.05). (**C**) Full length caspase-6 protein is cleaved in MCF-7 but not in T47D cells after treatment. Cells were treated with 1 μM STS for indicated times, lysates prepared and 50 μg protein subjected to SDS–PAGE followed by Western transfer and probed with caspase-6 antibody. C indicates untreated cells. The results are representative of three independent experiments.

DNA fragmentation is mostly mediated by caspase-3 cleavage of the inhibitory protein DFF45 resulting in release of the endonuclease DFF40 which is then free to cleave DNA (Enari *et al*, 1998; Wolf *et al*, 1999). It has been shown that MCF-7 cells lack caspase-3 protein. Caspase-3 expression was also absent in the MCF-7 cells used in this study, however we detected caspase-3 expression in T47D cells (data not shown). Therefore in contrast recent studies where lack of DNA fragmentation in response to TNFα and STS was reported (Janicke *et al*, 1998b), these results (Figure 3A

) show that despite the absence of caspase-3, DNA fragmentation was detectable in MCF-7 cells.

### Caspase involvement in staurosporine induced apoptosis of T47D and MCF-7 breast cancer cells

To examine whether caspases were involved in STS induced apoptosis, cells were pre-incubated for 1 h with z-VAD-fmk, a broad spectrum caspase inhibitor and were then treated for 16 h with 1 μM STS. As shown in Figure 3, z-VAD-fmk abrogated the apoptotic effect of STS in both T47D and MCF-7 cells. This demonstrates that STS-induced apoptosis requires activation of the caspase family of proteases. We then examined the activity of effector caspases firstly using the substrate DEVD which is the recognition site for caspases-3 and -7. There was no significant increase in DEVDase activity in MCF-7 cells at 12 and 24 h of treatment implying lack of involvement of caspase-7 since these cells do not express caspase-3 (Figure 3B). However, in T47D cells a time-dependent increase in DEVDase was observed from 12 h of treatment, which reaches maximal levels at 48 h implying involvement of caspase-3 and/or -7 in these cells (Figure 3B).

Effector caspase-6 was analysed by assessing disappearance of the full length protein by Western blotting. Levels of pro-caspase-6 protein did not alter in response to STS in T47D cells (Figure 3C). In contrast, in MCF-7 cells, pro-caspase-6 levels decreased following treatment with STS at 16 and 24 h (Figure 3C). Therefore, STS, appeared to induce a time dependent increase in caspase-3 or -7 activity in T47D cells and activation of caspase-6 in MCF-7 cells.

**Figure 4:**
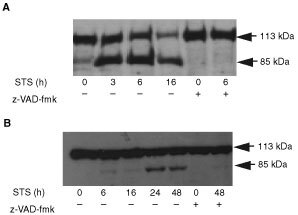
Staurosporine induced PARP cleavage in MCF-7 and T47D cells. Both (**A**) MCF-7 and (**B**) T47D cells were either treated with 1 μM STS for the times indicated or pre-incubated with 100 μM z-VAD-fmk for 1 h prior to STS treatments. Cell lysates (40 μg protein) were prepared and samples subjected to 8% SDS–PAGE followed by Western transfer and probed with PARP antibody (full length protein is 113 kDa, and cleaved fragment is 85 kDa). The results are representative of three independent experiments.

Activation of the caspase cascade leads to cleavage of cellular proteins such as the DNA repair enzyme poly-ADP-ribose polymerase (PARP); cleavage results in inactivation of the enzyme. STS induced PARP cleavage in a time-dependent manner in MCF-7 cells; the 85 kDa cleavage product was observed as early as 3 h after treatment and this increased dramatically by 16 h (Figure 4A

). PARP cleavage was abrogated on pre-treatment with z-VAD-fmk (Figure 4A). However, in T47D cells PARP cleavage was not detectable until 24 and 48 h of treatment, however only partial cleavage was evident; the level of full length protein 48 h after treatment was similar to that of control cells (Figure 4B). PARP cleavage was also inhibited by z-VAD-fmk in T47D cells.

### Staurosporine induces a reduction in the mitochondrial transmembrane potential (ΔΨm) and release of cytochrome *c* in T47D and MCF-7 human breast cancer cells

**Figure 5:**
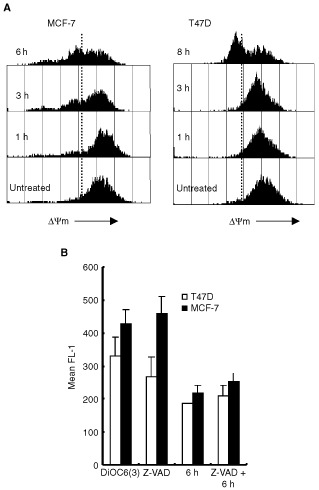
Alterations of the mitochondrial transmembrane potential (ΔΨm) in MCF-7 and T47D cells in response to STS. (**A**) Cells were treated with 1 μM STS for the times indicated. Cells were then stained with 40 nM DiOC_6_(3). Representative histoplots are shown for each time point. The dotted line separates cells with high and low fluorescence. (**B**) Caspase independent ΔΨm collapse. Cells were incubated with 100 μM z-VAD-fmk (Z-VAD) prior to 1 μM STS for 6 h. Mean fluorescence (FL-1) representing ΔΨm was measured by Flow Cytometry. Error bars represent the mean±s.e.m. of four independent experiments.

**Figure 6:**
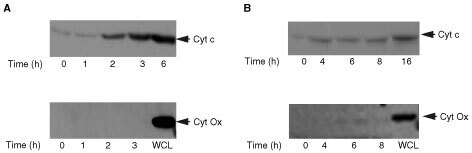
Staurosporine induced cytochrome *c* release into the cytosol of (**A**) MCF-7 and (**B**) T47D cells. After treatment with 1 μM STS for the times indicated cytosolic extracts were prepared and 30 μg protein subjected to 14% SDS–PAGE followed by Western blot analysis with anti-cytochrome *c* antibody (Cyt c) and cytochrome *c* oxidase subunit II (Cyt Ox). Whole cell lysate (WCL) was used as a positive control for the cytochrome *c* oxidase. Results are representative of four independent experiments.

Alterations in the mitochondrial transmembrane potential (ΔΨm) have been shown to be important for the release of mitochondrial proteins such as cytochrome *c*, which when in the cytosol can lead to activation of the caspase cascade and subsequent death (Reed, 1997). We measured the mitochondrial transmembrane potential (ΔΨm) by staining with 40 nM DiOC_6_(3) after treatment with 1 μM STS. STS caused a time-dependent reduction in the mitochondrial transmembrane potential (ΔΨm) in MCF-7 and T47D cells (Figure 5A

). In MCF-7 cells this was significant 3 h after treatment whereas significant changes were not observed until 8 h in T47D cells (Figure 5A). At each time point the majority of cells were PI negative implying this mitochondrial alteration occurs before any membrane changes. Moreover, Mitotracker Red staining at these time points was low and diffuse (data not shown), substantiating a reduction in ΔΨm after STS treatment. To verify that the breakdown of ΔΨm is an early event during STS induced apoptosis and was not subsequent to caspase activation, we tested the effect of z-VAD-fmk on the loss of ΔΨm. Cells were pre-incubated with z-VAD-fmk and then treated with 1 μM STS for 6 h. As shown in Figure 6B

, z-VAD-fmk had no effect on the reduction in ΔΨm at 6 h in either T47D or MCF-7 cells, suggesting this effect to be caspase-independent. To determine the effect of STS on cytochrome *c* release, cytosolic extracts were assessed by Western blotting. The results show that cytochrome *c* release was observed at 2 h after STS treatment in MCF-7 cells and 4 h after treatment in T47D cells (Figure 6A,B). Cytochrome *c* oxidase subunit II was used as a mitochondrial marker. Cytochrome *c* oxidase was not detected in any cytosolic extracts (Figure 6A,B) demonstrating no mitochondrial contamination. The data suggest that mitochondrial dependent mechanisms contribute to STS mediated apoptosis in breast cancer cells and that these events occur very early in the apoptotic pathway.

### Effect of Staurosporine on Bcl-2, Bcl-x_L_ Bak and Bax protein expression and Bax localisation in human breast cancer cells

There are numerous studies suggesting that overexpression of Bcl-2 or Bcl-x_L_ can block apoptosis by effects at the mitochondrion (Shimizu *et al*, 1996; Susin *et al*, 1997; Yang *et al*, 1997), whereas Bax and Bak have been shown to exert pro-apoptotic activity (Chittenden *et al*, 1995; Narita *et al*, 1998). We examined the effect of STS on the expression levels of Bcl-2, Bcl-x_L,_ Bak and Bax proteins. In MCF-7 and T47D cells STS did not alter Bcl-2, Bcl-x_L,_ Bax nor Bak protein expression from 3 to 24 h (MCF-7 cells) or 6 to 48 h (T47D cells) of treatment (data not shown). Bcl-2 expression in T47D cells was not detected by Western blotting, which is in agreement with a previous report (Zapata *et al*, 1998). Therefore STS mediated apoptosis in T47D and MCF-7 cells does not involve alterations in the expression of Bcl-2, Bcl-x_L_, Bak or Bax proteins.

**Figure 7:**
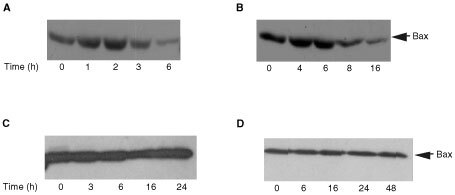
Staurosporine induced Bax redistribution from the cytosol to the mitochondria in breast cancer cells (**A**) MCF-7 (**B**) T47D. After incubation with 1 μM STS for the indicated times cytosolic extracts were prepared and subjected to 14% SDS–PAGE followed by Western blot analysis and probed with anti-Bax or anti cytochrome *c* oxidase subunit II antibody. Also shown are the Bax levels in the whole cell lysate of (**C**) MCF-7 and (**D**) T47D cells respectively. Results are representative of three independent experiments.

Bax translocation from the cytosol to the mitochondria has been demonstrated to occur in many cell lines in response to a variety of apoptotic inducers (Hsu *et al*, 1997; Gross *et al*, 1998). In order to determine the localisation of Bax in MCF-7 and T47D cells we exposed cells to 1 μM STS then prepared cytosolic fractions. As shown in Figure 7

, Bax protein levels decrease with time in the cytosolic extracts of MCF-7 and T47D cells, 3 and 8 h after treatment respectively. This is consistent with Bax translocating to the mitochondria after treatment with STS and that this occurs following the release of cytochrome *c* in both cell types, and also that this redistribution is an early event in these cells preceding PARP cleavage and caspase activation.

## DISCUSSION

Many chemotherapeutic drugs have been shown to induce apoptosis in various cancer types. It is therefore important to understand how each drug acts to induce apoptosis on certain cancers. Staurosporine has been shown to induce apoptosis in many cell types including, cardiomyocytes (Yue *et al*, 1998), chang liver cells (Swe and Sit, 1997), fibroblasts (Jacobson *et al*, 1996) and Jurkat T cells (Takahashi *et al*, 1997). Here we studied its effect and mechanisms of action on breast cancer cells. T47D and MCF-7 cells both underwent apoptosis in response to STS. MCF-7 cells were more sensitive to STS than T47D cells, and the onset of apoptotic events was different, with MCF-7 cells appearing to respond to STS induced apoptosis earlier. Maximal apoptosis was achieved 24 h after exposure to 1 μM STS in MCF-7 cells whereas this was 48 h in T47D cells. DNA fragmentation is an endpoint characteristic of apoptosis occurring after caspase activation. DFF45 is an inhibitor of an endonuclease DFF40. Once DFF45 is cleaved by caspases, DFF40 is then active and free to cause DNA fragmentation. Recent studies have suggested that caspase-3 and -7 and no other caspase so far identified are essential to cleave DFF45 and result in active DFF40 (Liu *et al*, 1999), and that caspase-3 was essential for DNA fragmentation in MCF-7 cells (Tang and Kidd, 1998; Zapata *et al*, 1998). However, as the MCF-7 cells used in the present study did not express caspase-3 protein, the possibility existed that caspase-7 might substitute for caspase-3. Indeed, our results show DNA fragmentation in both cell types was completely inhibited by z-VAD-fmk implying this effect to be caspase mediated. However, DEVDase activity was not detected in MCF-7 cells in response to STS suggesting that caspase-7 is not involved. Therefore, we suggest that in MCF-7 cells another mechanism exists, which is a caspase-3 independent pathway causing the DNA fragmentation observed in this study. Many studies have utilised MCF-7 cells to study apoptosis and have demonstrated DNA fragmentation in response to many stimuli (Saunders *et al*, 1997; Hishikawa *et al*, 1999; Tudor *et al*, 2000). For example, nitric oxide induced apoptosis in MCF-7 cells with a lack of DEVDase activity substantiating the suggestion for an alternative mechanism in MCF-7 and possibly other cells that do not express caspase-3 (Umansky *et al*, 2000). More recently it has been demonstrated that in the absence of caspase-3 in MCF-7 cells, DNA fragmentation did not occur and cytochrome *c* release was slower compared to caspase-3 expressing cells (Blanc *et al*, 2000). Also caspase-9 processing was impaired in response to cisplatin in MCF-7 cells. Such different findings to our results could be due to the difference in the variants of MCF-7 cells used. There have been many studies highlighting the differences in MCF-7 cell variants (Osborne *et al*, 1987; Burow *et al*, 1998; Gooch and Yee, 1999) by illustrating the differences in DNA fragmentation in response to a variety of apoptotic stimuli. Yet it is clear that, in the absence of caspase-3, MCF-7 cells do undergo caspase-3 independent DNA fragmentation. Indeed, caspase-3 −/− hepatocytes and thymocytes do display DNA fragmentation although, with delayed kinetics compared to caspase-3 +/+ cells (Zheng *et al*, 1998). This also points to an alternative mechanism independent of DFF45/DFF40 in caspase-3 deficient cells. In this study, DNA fragmentation was inhibited by z-VAD-fmk implying that another caspase is involved. Recent cloning of DFF45/ICAD homologues (Inohara *et al*, 1998) raise the possibility that other DFF45/DFF40 complexes exist which can be activated independently of caspase-3. Indeed, in support of this idea it has been demonstrated that, in the absence of caspase-3, hepatocyte cells induce compensatory caspase activation, such as caspase-6 (Zheng *et al*, 2000). This could also be the case for MCF-7 cells, as we have shown that caspase-6 is activated in the absence of caspase-3 and may therefore illustrate redundancy within the caspase family of proteases. Although caspase-6 processing was detected in MCF-7 cells, full length protein was still present at 24 h. Caspase-6 processing has been shown to require caspase-9 and caspase-3; therefore in the absence of caspase-3 this could explain why partial processing was observed. In T47D cells, DEVDase activity was detected implying the classical pathway leading to cleavage of DFF45 and subsequent DNA fragmentation occurs. In addition, T47D cells displayed typical apoptotic morphology yet in MCF-7 cells apoptotic body formation was absent. Apoptotic body formation involves p21 activated kinase (PAK2) which when cleaved becomes constitutively active and has been demonstrated to be a substrate of caspase-3 (Rudel and Bokoch, 1997). In the absence of caspase-3, PAK2 would not be activated and this could account for the abnormal apoptotic morphology in MCF-7 cells. Proteolytic processing of PARP was detected at 3 h in MCF-7 cells yet changes in caspase-6 were not observed until 16 h. However, release of cytochrome *c* into the cytoplasm leads to the formation of an apoptosome, which is a complex of dATP, cytochrome *c*, Apaf-1 and procaspase-9 that results in activation of caspase-9. PARP is also a substrate for caspase-9 therefore the activation of caspase-9, could account for the early cleavage of PARP in MCF-7 cells. In T47D cells only partial PARP cleavage was observed at 24 and 48 h of treatment which was coincident with DEVDase activity in these cells.

Mitochondrial changes were observed in both MCF-7 and T47D cells but again with different kinetics. We have demonstrated cytochrome *c* release from the mitochondria to the cytosol in both MCF-7 and T47D cells at 2 and 4 h respectively. Cytochrome *c* release from mitochondria that occurs after STS treatment is possibly controlled by Bax. Bax has been shown to be cytosolic and in association with intracellular membranes including the mitochondrial membrane, but upon apoptotic stimuli Bax inserts into the mitochondrial membrane and leads to the release of cytochrome *c* (Goping *et al*, 1998). It has also been shown that Bax redistribution occurs in cells at different times (Wolter *et al*, 1997). In MCF-7 and T47D cells, Bax protein levels declined in the cytosol at 3 and 8 h respectively after treatment, which was subsequent to cytochrome *c* release. It is therefore possible that, in these cells, sufficient Bax resides at the mitochondrial membrane to induce cytochrome *c* release after a death signal, and further apoptotic insult leads to increasing amounts of Bax translocating to the mitochondria. This could explain why we do not observe significant changes in Bax protein translocation until 3 and 8 h after STS exposure, although it does not rule out Bax being responsible for cytochrome *c* release. In addition to triggering cytochrome *c* release, Bax induces a reduction in the mitochondrial transmembrane potential (ΔΨm). This was observed at 3 h in MCF-7 cells and 6 h in T47D cells, and occurred coincident with significant Bax translocation. In both cases, the release of cytochrome *c* into the cytosol preceded the loss of ΔΨm, which is consistent with a caspase independent mechanism upstream of the mitochondria. Loss of ΔΨm has been postulated to involve opening of a large permeability transition pore (PTP). This would suggest that in MCF-7 and T47D cells cytochrome *c* release occurs independently of PTP opening.

Taken together, our data demonstrate that the mechanism of STS mediated activation of different caspase cascades in T47D and MCF-7 cells involved the release of cytochrome *c*, loss of ΔΨm and Bax redistribution. Interestingly, despite the lack of caspase-3, MCF-7 cells proceeded to apoptosis more rapidly. Also, the lag time between the earliest event observed (cytochrome *c* release) and morphological and biochemical evidence of apoptosis (ISNT, annexin V) was longer in T47D cells (around 8 h) than in the caspase-3 deficient MCF-7 cells (around 1–2 h). This study provides further evidence for the existence of another mechanism of DNA fragmentation in the absence of caspase-3. However, we have also shown that two similar tumour types undergo STS induced apoptosis with similar outcomes, but with very different kinetics, and emphasising that pathways to apoptosis can be very different in similar cell types. Such variations may have relevance to tumour progression in a clinical setting since cell may respond differently to chemotherapy, and perhaps also to survival factors.
